# Factors that lead to the use of crack cocaine in combination with marijuana in Brazil: a qualitative study

**DOI:** 10.1186/s12889-015-2063-0

**Published:** 2015-07-25

**Authors:** Janaina R. Gonçalves, Solange A. Nappo

**Affiliations:** Department of Preventive Medicine, Brazilian Center of Information on Psychotropic Drugs (CEBRID), Universidade Federal de São Paulo, Rua Botucatu, 740, CEP 040-23062 São Paulo, Brazil

**Keywords:** Crack cocaine, Cannabis, Crack cocaine and cannabis association, Brazil

## Abstract

**Background:**

In Brazil, crack cocaine use remains a healthcare challenge due to the rapid onset of its pleasurable effects, its ability to induce craving and addiction, and the fact that it is easily accessible. Delayed action on the part of the Brazilian Government in addressing the drug problem has led users to develop their own strategies *for* surviving the effects of crack cocaine use, particularly the drug craving and psychosis. In this context, users have sought the benefits of combining crack cocaine with marijuana. Our aim was to identify the reasons why users combine crack cocaine with marijuana and the health implications of doing so.

**Methods:**

The present study is a qualitative study, using in-depth interviews and criteria-based sampling, following 27 crack cocaine users who combined its use with marijuana. Participants were recruited using the snowball sampling technique, and the point of theoretical saturation was used to define the sample size. Data were analyzed using the content analysis technique.

**Results:**

The interviewees reported that the combination of crack cocaine use with marijuana provided “protection” (reduced undesirable effects, improved sleep and appetite, *reduced craving* for crack cocaine, and allowed the patients to recover some quality of life).

**Conclusions:**

Combined use of cannabis as a strategy to reduce the effects of crack exhibited several significant advantages, particularly an improved quality of life, which “protected” users from the violence typical of the crack culture.

Crack use is considered a serious public health problem in Brazil, and there are few solution strategies. Within that limited context, the combination of cannabis and crack deserves more thorough clinical investigation to assess its potential use as a strategy to reduce the damage associated with crack use.

## Background

The use of crack cocaine emerged in Brazil in the early 1990s in the city of São Paulo [1], and the first drug seizure made by the Civil Police of São Paulo in 1991 although crack cocaine was reportedly present in the country as early as 1989 [2]. The status of healthcare in Brazil at that time was extremely worrisome. An AIDS epidemic was ravaging the country, particularly in the state of Sao Paulo, where people who use injection drugs (IDUs) constituted one of the groups susceptible to viral infection due to the route of drug administration [3]. The majority of government and healthcare professional attention was focused on this situation [4, 5]. Because of this serious public health problem and the emergence of crack cocaine, several IDUs started to use this new drug in an attempt to protect themselves from the threat of contracting the virus via an intravenous route of administration [6]. This change in the administration route was not a very difficult step given that crack cocaine offered some “advantages”; it was inexpensive, its “powerful” effects were achieved in a matter of seconds, it was easily administered, and it was considered to be a “clean” drug because it did not have the potential to transmit HIV or other STDs due to the administration route [6, 7]. Initially, the use of crack cocaine functioned as an informal harm reduction strategy. According to Mesquita et al. [8], the transition in administration route may have contributed to reducing HIV infection rates during the period from 1991 to 1999, which decreased from 63 to 42 % in the city of Santos, the city with the highest rate of HIV infection. At that time, crack cocaine use was not a problem that attracted the attention of the authorities, although there were already reports of its devastating effects [6]. The establishment of the Brazilian “cracolandias” (“crack lands”) - public places where crack cocaine users met to consume the drug out in the open - was also not sufficient to elicit more rapid action from the state regarding crack cocaine use [9]. Perhaps as a result of the scenario at that time (the AIDS epidemic), the State’s actions in confronting the drug problem were delayed, leading crack cocaine users to devise their own solutions to the problems arising from their use of this drug. The potential for harm caused by crack cocaine is quite high when the negative outcomes produced are considered, making its abuse a public health problem in Brazil today [10]. The harmful effects of crack cocaine include the high degree of disintegration of socioeconomic and mental health; the intense involvement with crime, marginalization, violence, prostitution, and multiple sexual partners; and the consequent increased potential for HIV infection [11–14].

Ribeiro et al. [15] examined the strategies developed by users and found that they employed simple strategies, such as using the drug in protected locations; avoiding visibility; fulfilling commitments with drug traffickers to avoid reprisals, including death; staying quiet in the vicinity of the “bocada” (drug trafficking area) and not arousing the attention of the neighborhood and the police; and combining other drugs, such as marijuana or alcohol, with crack cocaine use. Chaves et al. [16] investigated the strategies employed by crack cocaine users to control their craving and observed strategies similar to those used in the study mentioned above; however, they also noted the combination of crack cocaine use with other drugs, especially marijuana and alcohol.

The use of marijuana seems to be common among crack cocaine users; however, few studies have considered the user’s opinions in addressing this phenomenon.

The current study used a sample that was selected based on the criteria for purposeful sample, exhibiting appropriate level of rigor for satisfactory qualitative study. However, the most common combination mentioned by other authors involved adding crack rocks to marijuana inside of a cigar [17]. However, in the present study, additional cannabis combinations were investigated.

In this context, the approach used in the present study was justified (i.e., the users themselves reported the “advantages” and disadvantages of this combination based on their knowledge, values, and points of view, allowing for an understanding of their reality).

## Methods

A qualitative methodology was used because it allowed us to analyze study participants’ beliefs about the combination of marijuana and crack cocaine based on their own views and concepts [18–20].

### Sample recruitment

Seven key informants (KIs) were selected during the first phase of the study - five psychiatrists and two psychologists – who had varied knowledge about the study topic and the study population [20]. These KIs were invited for an informal conversational interview without a previously prepared script. Relevant questions regarding the topic arose in the context of these conversations [21–23]. The interviews were recorded, transcribed, and analyzed, and the data generated was then used by the researchers to prepare an interview script that was used with the study participants (crack cocaine users) [20]. Due to difficulties in accessing the study population due to the illegality of crack cocaine use, some of the KIs also played a gatekeeper role (i.e., they provided access to the study participants) [18]. Because the gatekeepers were known by the study population, they inspired the trust of the drug users, facilitated the participation of that population in the study, and were the first point of contact between the study population and the researchers. Each KI identified potential participants and discussed the study with them prior to introducing the researchers. Those who agreed to participate were instructed to contact the researchers. In-depth interviews were conducted using purposeful sampling, the components of which followed particular criteria (criterion sampling) [20]: Crack cocaine users who were older than 18 years of age and who had combined crack cocaine with marijuana use a minimum of 25 times, thereby ensuring that experimental users were not included in the sample [24]. These criteria led to a sample size of 27 participants, all of whom were selected in the city of São Paulo during the years 2012–2013. The first interviewees who were contacted by the KIs identified other possible participants, thereby using the snowball technique to compose the sample. Sampling using the snowball technique starting with the first interviewee created a chain of interviewees [23, 25]. To include the largest possible number of user profiles in the sample who met the inclusion criteria proposed, various chains of interviewees were sought, and seven chains were identified, which ranged from 3 to 5 individuals each. The sample size was adequate to cover all of the topics of interest and various user profiles. This assumption was met when the interviewees’ responses became redundant. At this point, termed the theoretical saturation point, the lack of new information and the repetition of responses were identified [18, 20, 23].

### Instruments used

Semi-structured interviews were conducted using a script of topics that were selected based on the information provided by the KIs [20, 22]. The script was composed of previously standardized questions to facilitate comparisons among responses and reduce interviewer interference. Additional questions emerged to clarify specific topics during each interview, allowing for improvement of subsequent understanding [22, 23]. The script consisted of socio-demographic data, history of drug use, history of crack cocaine use, associated marijuana use, and damages/“advantages” of the combination. The questions relating to the socioeconomic data were evaluated using the *Brazilian Economic Classification Criteria 2008* scale, published by the ABEP (Brazilian Association of Research Companies-Associação Brasileira de Empresas de Pesquisa) [26]. This scale mainly considers the consumer goods possessed by the family and classifies respondents into classes A1, A2, B1, B2, C, D and E (A1 is the category with the greatest ownership, whereas E delineates a lack of ownership and includes the homeless). The criteria for dependency, as defined by the Diagnostic and Statistical Manual of Mental Disorders (DSM-IV) [27], were also incorporated into the script. After obtaining the consent of the interviewees, the interviews were recorded, each of which lasted approximately 70 min.

### Qualitative analysis of the content

Each interview was identified by an alphanumeric code in which the first letter was the first initial of the interviewee’s name, followed by his or her age and gender. The interviews were transcribed and reviewed by the researchers. They were then analyzed using the content analysis technique based on Bardin’s theoretical framework [28]. The various parts of the interviews were split and then grouped according to each research theme. For the analysis, NVivo Software Version 10 was used, which allowed for greater data analysis consistency and facilitated organization. The importance of the themes identified was analyzed by considering the emic approach. This step, defined as categorization, was developed by the two researchers who independently and simultaneously analyzed the data. Then, these two analyses were compared to obtain consistency and coherence in the results. Finally, inferences supporting the explanations were initiated and conclusions were generated.

Q*uotes* from the interviewees’ statements are presented in the results section; they are identified by their code and shown in italics.

### Ethical consideration

The study protocol was approved by the Ethical Review Committee of the Federal University of São Paulo (CEP 1602/11). In the study, oral informed consent was obtained from each participant at the beginning of the initial interview after they were given information about the study and informed that they could withdraw at any time. With permission, interviews were recorded using a digital recorder and later transcribed in full. Anonymity of participants was maintained.

## Results

### Characteristics of the sample

The sample, which contained 27 crack cocaine users between the ages of 19 and 49 years (mean 25.9), consisted mostly of 21- to 30-year-old males who had received little education (only elementary school), belonged to a low social class (class E) [26], were unemployed, were living in shelters or on the street without family and were crack cocaine*-*dependent*.* All of the participants proved to be crack cocaine*-*dependent according to DSM-IV [27] criteria, and nearly 70 % of them were also reported to be marijuana*-*dependent.

Drug use was not always recreational. All of the participants in the sample reported having had problems with some of the drugs cited, but crack cocaine was the drug that most affected all aspects of their lives. Social problems, such as robberies and loss of family, job, and social status, were most commonly cited, followed by physical injuries resulting from assaults, impaired appearance, etc. Cravings and transient paranoid symptoms were described as the effects of the drug that most contributed to these damages.

The participants reported having used various self-devised strategies to either stop crack cocaine use or overcome the problems caused by it, including seeking help in religion, avoiding contact with crack cocaine users, and the use of other drugs combined with crack cocaine.

### Combination with other drugs

According to the interviewees’ statements, the combination of crack cocaine and marijuana was not the only drug combination used. Even sporadically, drugs, such as alcohol, hallucinogens (*Ecstasy*), and snorted cocaine, and medications, such as benzodiazepines, were also used in combination with crack cocaine in an attempt to either increase the pleasurable effects or minimize the unpleasant effects. However, as with the combination of crack cocaine and marijuana, the combination with alcohol was also mentioned often.

The criteria used to choose a drug to be combined with crack cocaine were based on the experience of other users, experimentation, and the individual evaluation of the effects of the combination tested.

### Reasons cited for combining crack cocaine with marijuana^1^

Based on the interviewees’ statements, it became evident that in the context of crack cocaine use, the participants attributed a “protection” role to marijuana that was revealed in several ways.

Reduction of unpleasant effects: The participants attributed relaxing properties to marijuana, which interfered with the effects of crack cocaine by decreasing those effects that were considered to be undesirable. Additionally, participants considered that the effects resulting from the combination were pleasant. Transient paranoid symptoms, which are a typical effect of crack cocaine use and according to users can cause fear, distrust, and sometimes violent behavior, were the effects that were most commonly mentioned by the interviewees as being suppressed in the presence of marijuana.

*When I mix marijuana with crack, the effect of marijuana is stronger than the effect of crack cocaine, which then curbs paranoia* (psychosis) *and controls cravings* (G38MC).

*The “mesclado”* (“mix”) *makes me fly. It takes away the wickedness of the rock. I do not look for pieces of rock on the ground, like a fool. The effect of the “mesclado” is better; it is very different* (V43MC).

Reduction of crack cocaine-seeking behavior: The serenity caused by marijuana helped the participants control their cravings so that the *desire to smoke was reduced*. Because of this effect, strategies to obtain the drug, such as thefts and robberies, did not need to be used, which protected the users from possible fatalities resulting from these activities. The participants stated that marijuana caused a type of “numbness” of the mind, which made them “forget” about crack cocaine, even if only temporarily. The focus on crack cocaine was displaced by the effects resulting from the combination.

*I am alert to everything, but then the effect of marijuana makes me feel calm and I do not think about stealing. I do not think about doing something wrong. I just stay there enjoying that effect, understand?* (G38M)

Reduction of aggressiveness: This issue was highlighted as having a marked effect on the crack cocaine use culture. When asked about the influence of marijuana on this effect, the vast majority of participants stated that there was a reduction in, or even absence of, aggressiveness.

*The pure rock makes me aggressive because I want to smoke more, and if someone stops me from doing this, I become violent, even if it is a family member. With marijuana, I control this situation because I am more relaxed, less anxious.* (G24M)

#### Quality of life

Participants also reported that the combination of the two drugs allowed for the partial recovery of the quality of life that was lost with crack cocaine use. Basic human needs that were previously compromised by the use of crack cocaine were regained with the use of marijuana combined with crack cocaine. Sleep, hunger, and sex are examples of this well-being that were cited by the interviewees.

…*with marijuana, I sleep well. I eat well. I have good sex. It makes me calm; the same feeling as taking diazepam, a tranquilizer. I become more cool* (nice)*…* (V49M)

#### Savings

According to some interviewees, mixing crack cocaine with marijuana helped them to save money to buy crack cocaine because a portion of their crack cocaine use was replaced by marijuana use. According to the participants’ reports, this resulted in a higher crack cocaine yield.

*It lasts longer. Assuming that I smoke a crack cocaine rock that costs 10 Brazilian reais* (approximately $5) *in a half hour, with marijuana, it will take one hour. I split it into small bits, I stop, and then I smoke more.”* (R27M)

The combination of crack cocaine with marijuana was not always perceived as beneficial by the user. Those who disliked the combination of crack cocaine with marijuana attributed this dislike to the reasons outlined below.

Decrease in the potency of the drug: A small number of participants did not consider the combination of crack cocaine with marijuana to be beneficial; specifically, the action of marijuana on the effects of crack cocaine (i.e., reducing its intensity) was not well accepted by everyone. The calm and relaxation promoted by the combination that represented a gain for some of the participants was the cause of displeasure for others, so that the participants started using the “mesclado”, but after a period of time, they went back to smoking crack cocaine alone, without combining it with marijuana.

*I lost interest because the “mesclado” makes the person much more quiet… And I do not want to be quiet, not in that way… so I tried the pure crack cocaine and found that the pure crack cocaine made me more alert…* (P34M)

Undesirable effects: Participants reported the occurrence of undesirable psychological effects when marijuana was used, reinforcing the idea that drug use is an individual experience.

*If I smoke some marijuana, I do not feel good. I feel depressed. I feel very bad. I think it is because I used too much crack, so it affected my brain a bit. If I smoke a joint today, I feel worse than if I had smoked a rock. I feel very depressed.* (M23F)

### The sequence of the marijuana and crack cocaine combination

The sequence of the marijuana and crack cocaine combination seemed to be of great importance in the effects resulting from combining the two drugs. Some of the participants preferred to use marijuana before smoking crack cocaine, whereas others preferred to use it after smoking crack cocaine, but the simultaneous use of marijuana and crack cocaine was most commonly observed in this sample (“mesclado” or “pitilho”).

### Simultaneous use of marijuana and crack cocaine (mesclado)

As mentioned previously, the “mesclado” was the preferred manner of combining these two drugs. The participants attributed this preference to several factors, which are outlined below.

Does not attract attention on the street: According to the interviewees, smoking from a pipe makes crack cocaine use obvious; they are identified as users anywhere they use a crack pipe. Using a cigarette, in which marijuana is mixed with the rock (crack cocaine), is more “protective”. These users face no discrimination because they are not identified as crack cocaine users (known as a “craqueiro” in Brazil). Additionally, some of the interviewees stated that the unpleasant effects, mainly the transient paranoid symptoms, disappeared, and the combined use of marijuana and crack cocaine also helped them to remain calmer, which contributed to greater social acceptance and consequently decreased the high degree of marginalization to which they were subjected.

*I can smoke in a park without any problem. Using only the rock, I get paranoid* (psychotic)*, suspicious of everyone. I just want to be hidden from view. I think about stealing all the time.* (R31M)

The interval between consumption is increased: The interviewees stated that when they smoked the “mesclado”, it took longer for them to want to smoke it again, resulting in them consuming a smaller amount of crack cocaine. With the “mesclado”, 1 to 2 h elapsed before they repeated the drug use; in contrast, when they used only crack cocaine, this period of time was reduced to 5 or 10 min.

### Use of marijuana before crack cocaine

A few interviewees reported using marijuana before crack cocaine because they claimed that in such circumstances, they became more relaxed and calmer and would not consume crack cocaine afterwards while they experienced this tranquility. Some of the interviewees revealed that the only way to change this condition was to consume alcohol to “break” this state and then return to smoking crack cocaine after marijuana use. These implications led them to stop using marijuana in this manner, replacing it with other manners of use, the most common of which was the “mesclado”.

### Use of marijuana after crack cocaine

Marijuana was used in this sequence with the same goal as that of the previous sequence, which was the reduction of the undesirable effects of crack cocaine. Those who consumed marijuana after crack cocaine reported that it prevented them from seeking more crack cocaine to continue using it.

*I smoked it [marijuana] afterwards to stop, understand? Because after the effect of marijuana… that was it! Then, I was relaxed.”* (C33M)

Some users reported a slightly different goal: after they had consumed all of the crack cocaine and did not have the possibility of getting more of the drug, they smoked marijuana to abolish the desire to smoke more crack.

It is important to highlight that in this sample, combinations with other drugs were aimed at reducing the undesirable effects of crack cocaine and allowing the users to smoke it in a calmer manner. Accordingly, with the exception of one user, none of the participants attempted to replace crack cocaine with another drug (in this case, marijuana). Despite the positive results promoted by the use of marijuana before crack, some of the interviewees altered the sequence of this combination due to the unfavorable environment created by this drug, which made the use of crack cocaine more difficult.

## Discussion

The present study examined the combination of crack cocaine and cannabis as an informal alternative to cope with the use of crack cocaine. This combination has been previously mentioned in other studies by Brazilian authors [15–17, 29].

The study sample consisted mainly of young men of low socioeconomic status, with little schooling, who were living on the street. These characteristics are consistent with the profile of Brazilian crack users recently described by the government [30]. Thus, the study findings are likely reflective of the broader population of crack users in Brazil given the similarities in characteristics noted here.

In their narratives, the interviewees described the benefits of the cannabis-crack combination. A reduced craving, which is considered the main cause of dependence and involvement in high-risk situations to obtain the drug [16], was one of the advantages most often mentioned by the participants. In a study conducted with a convenience sample of six users undergoing treatment, Andrade et al. [17] found that a reduced craving was the greatest benefit of using the crack-cannabis combination. Participants in the present study additionally reported a reduction of transient paranoid symptoms, which sometimes lead to violence [31, 32], as a positive outcome of the combination.

The interviewees emphasized that the improved quality of life as a result of eliminating or reducing cravings and paranoid symptoms was the most positive effect of using the cannabis-crack combination. This effect protected them from marginalization and/or violent environments, which are the main causes of death, favoring the reduction of their marked vulnerability in the crack cocaine use culture [10]. Additionally, a persistent relationship with society is highly valuable in terms of potential access to healthcare services, which might help improve their wellbeing [33]. This combination also resulted in decreasing crack cocaine-seeking behavior, which in turn reduced use of crack cocaine, increased monetary savings, and increased survival. The unhealthy appearance of crack cocaine users due to drug-induced appetite inhibition was compensated for by marijuana use, which awakened the appetite, causing weight gain, and also improved sleep [34].

The most common combination mentioned by other authors and the present study participants involved adding crack rocks to marijuana inside of a cigar [17]. However, in this study, they also reported other methods of drug intake, such as using cannabis before or after crack. In either case, the interviewees slowed or even stopped their crack use due to the state of relaxation induced by cannabis. It is worth noting that participants were not always pleased with the outcome because their focus was not on quitting crack. Nevertheless, these types of combinations should be given more attention in strategies based on the replacement of drugs associated with multiple physical and mental complications by less damaging drugs.

The therapeutic effects of cannabis have been known for a long time [35]. Carlini et al. [36] and Leite and Carlini et al. [37], in the 1980s, demonstrated the medicinal properties of marijuana, especially the anticonvulsant properties of the drug.

Webb et al. [38] observed 100 patients who were using cannabis for medicinal purposes, and the authors obtained significant results, including the fact that 50 % of the patients experienced reduced levels of stress and anxiety, 45 % experienced improved sleep, and 12 % experienced improved appetite. Brunt et al. [39] also confirmed these beneficial effects of cannabis.

These studies provide support for the action of marijuana on the effects of crack cocaine. For example, the transient paranoid symptoms characteristic of crack cocaine use are suppressed by a component of *cannabis* called *cannabidiol* that has effective antipsychotic properties [40–42]; furthermore, the hypnotic effects of components of *cannabis* account for the stabilization of sleep in the user, which is extremely impaired with the use of crack cocaine [43].

However, the benefits of the use of marijuana combined with crack cocaine do not occur without consequences. Whereas most of the participants emphasized the benefits afforded by the cannabis-crack combination, it was clear that the intensity of the pleasurable stimulating effects of crack were decreased with cannabis use. This effect prevented wider acceptance of the strategy and led some users to abandon the method. Another notable fact is that 70 % of the sample met the DSM-IV criteria [27] for cannabis dependence, which must be taken into consideration in assessing the risk/benefit ratio of the cannabis-crack combination.

The value of this combination is difficult to evaluate and discuss in detail due to the illegality of cannabis in Brazil [44]. This fact has prevented important advancements from being made in the investigation of whether cannabis could be used as an alternative to reduce the damage caused by the abuse of and dependency on crack cocaine [45].

## Conclusion

Combined use of cannabis as a strategy to reduce the effects of crack exhibited several significant advantages, particularly an improved quality of life, which “protected” users from the violence typical of the crack culture.

Crack use is considered a serious public health problem in Brazil, and there are few solution strategies. Within that limited context, the combination of cannabis and crack deserves more thorough clinical investigation to assess its potential use as a strategy to reduce the damage associated with crack use.

### Study limitations

This study was a preliminary study with a qualitative approach. The study sampled 27 participants and was not representative of the total population of crack users in Brazil.

### Study strengths

Given the serious and growing problem posed by crack use in Brazil and the few possible solutions available, the benefits of combined cannabis and crack demonstrated in the present study should not be dismissed. The results of the present study could aid in emerging discussions in Brazil on the therapeutic properties of marijuana.

## Footnotes

1 This combination, referring to when the two drugs are used together, was given various names in different regions of the country. In the southeast, it was known as “mesclado” (mix), and in the northeast, it was known as “pitilho”).
